# CDKD-w+: A Keyframe Recognition Method for Coronary Digital Subtraction Angiography Video Sequence Based on w+ Space Encoding

**DOI:** 10.3390/s25030710

**Published:** 2025-01-24

**Authors:** Yong Zhu, Haoyu Li, Shuai Xiao, Wei Yu, Hongyu Shang, Lin Wang, Yang Liu, Yin Wang, Jiachen Yang

**Affiliations:** 1 School of Electrical and Information Engineering, Tianjin University, Tianjin 300072, China; yongzhu@tju.edu.cn (Y.Z.); li_hy18@tju.edu.cn (H.L.); yangjiachen@tju.edu.cn (J.Y.); 2 Tianjin Institute of Software Engineering, Tianjin 300387, China; yuwei@tjise.edu.cn (W.Y.); hyshang@tjise.edu.cn (H.S.); wanglin@tjise.edu.cn (L.W.); liuyang@tjise.edu.cn (Y.L.); wy@tjise.edu.cn (Y.W.)

**Keywords:** digital subtraction angiography, heartbeat keyframe localization, coronary DSA image encoding, X-ray sensor images

## Abstract

Currently, various deep learning methods can assist in medical diagnosis. Coronary Digital Subtraction Angiography (DSA) is a medical imaging technology used in cardiac interventional procedures. By employing X-ray sensors to visualize the coronary arteries, it generates two-dimensional images from any angle. However, due to the complexity of the coronary structures, the 2D images may sometimes lack sufficient information, necessitating the construction of a 3D model. Camera-level 3D modeling can be realized based on deep learning. Nevertheless, the beating of the heart results in varying degrees of arterial vasoconstriction and vasodilation, leading to substantial discrepancies between DSA sequences, which introduce errors in 3D modeling of the coronary arteries, resulting in the inability of the 3D model to reflect the coronary arteries. We propose a coronary DSA video sequence keyframe recognition method, CDKD-w+, based on w+ space encoding. The method utilizes a pSp encoder to encode the coronary DSA images, converting them into latent codes in the w+ space. Differential analysis of inter-frame latent codes is employed for heartbeat keyframe localization, aiding in coronary 3D modeling. Experimental results on a self-constructed coronary DSA heartbeat keyframe recognition dataset demonstrate an accuracy of 97%, outperforming traditional metrics such as L1, SSIM, and PSNR.

## 1. Introduction

Currently, deep learning technologies are developing rapidly, giving rise to various methods such as reinforcement deep learning [1], semi-supervised learning [2], active learning [3], transfer learning [4], federated learning [5], etc., which can be applied in multiple fields such as autonomous driving [6], path planning [7], image quality assessment [8], image super-resolution [9], smart healthcare [10], Internet of Things [11], image generation, generated image detection [12,13], and more. In the medical field, various deep learning applications such as image classification [14], object detection [15], image segmentation [16], patient privacy protection [17], and 3D modeling [18] can assist in medical diagnoses, helping medical personnel improve diagnostic efficiency and accuracy.

Digital Subtraction Angiography (DSA) is a medical imaging technique used to visualize vascular structure and evaluate blood flow within blood vessels. It is an important tool for diagnosing coronary heart disease, determining coronary stenosis, and performing cardiac intervention therapy. The basic principle of DSA is to use X-ray sensors to obtain a series of images before and after injecting contrast agents, and then use digital processing techniques to subtract these two sets of images, leaving only the vascular image containing the contrast agent, thus clearly displaying the morphology and blood flow of the blood vessels, and obtaining a two-dimensional image of the coronary artery of the heart. Coronary artery DSA is performed during surgery, and doctors can obtain two-dimensional images of the coronary artery from any angle by adjusting the camera position. Due to continuous shooting during this process, multiple images from that angle can be obtained, and doctors can understand the condition of coronary artery lesions from different camera positions and images at different times. However, due to the complexity of the structure of the heart and coronary arteries, two-dimensional images sometimes struggle to provide sufficient information, especially in assessing the morphology and severity of coronary artery lesions. Additionally, the repeated use of X-ray sensors for X-ray irradiation poses potential risks to the health of both doctors and patients. For this, three-dimensional modeling of coronary DSA can be performed. 3D modeling of coronary artery DSA images can help doctors gain a more intuitive and comprehensive understanding of the three-dimensional structure and lesion situation of the coronary artery, enabling more accurate localization and evaluation of stenosis, thus better determining whether intervention treatment is needed and selecting the most suitable treatment strategy [18].

**Figure 1:**
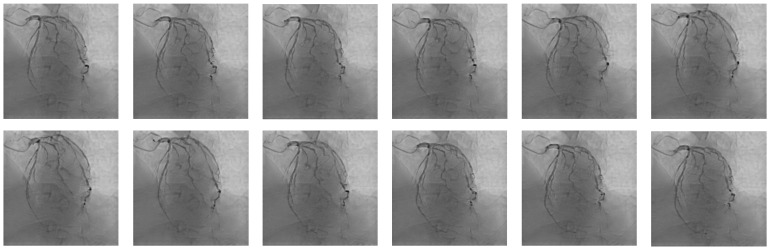
A DSA sequence containing a complete cardiac cycle.

**Figure 2:**
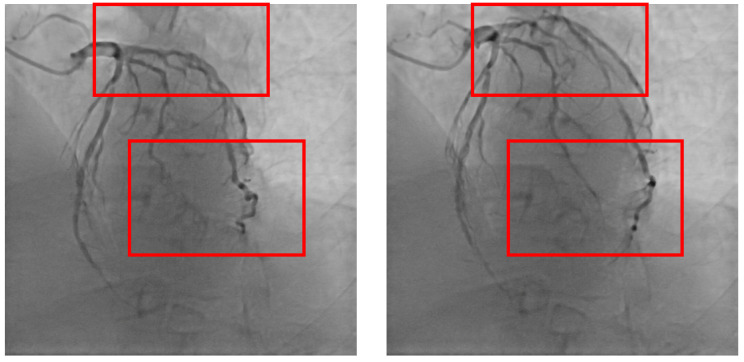
An intuitive display of the vasodilation and vasoconstriction frames in Figure 1, with marked positions indicating significant differences.

At present, the main method for 3D modeling of coronary arteries in the medical field is CT, but not all patients use both DSA and CT during treatment. In addition, the accuracy of CT images is lower than that of DSA images, so the modeling accuracy is not sufficient. In order to solve the problem of 3D modeling of DSA images, the main method currently used in academia is based on mathematical formulas and the principle of DSA imaging. By reconstructing blood vessels based on images from multiple camera positions, it is necessary to manually locate key points for offset correction. In recent years, methods for 3D modeling based on deep learning have developed rapidly, and new perspective synthesis methods represented by neural radiance field (NeRF) [19] can generate new camera-level views, demonstrating high potential in the field of 3D modeling of coronary DSA images. However, directly applying methods such as NeRF to 3D modeling of coronary artery DSA images has poor results. This is because the heart continues to beat, resulting in varying degrees of contraction and relaxation of the coronary arteries. There are significant deviations between DSA angiography image sequences. If images from different positions and states are used simultaneously to 3D model the coronary artery, it will introduce significant errors, leading to the inability of the 3D model to accurately reflect the coronary artery. As shown in Figure 1 and Figure 2, there are significant differences in coronary arteries between different frames of the same DSA sequence. To solve this problem, the heartbeat keyframes of the coronary DSA image sequence can be located, and the images of the heart contracting or relaxing to the poles can be selected from various positions. The images at this time node can be used as the basis for coronary 3D modeling, thus completing the registration of heartbeat states at different positions and achieving accurate coronary modeling. We propose a coronary DSA heartbeat keyframe recognition method CDKD-w+ (Coronary DSA Keyframe detection based on w+ space features) for this purpose. This method uses the pixel2style2pixel (pSp) [20] module to encode coronary DSA images in the high-dimensional space proposed by StyleGAN2, obtaining the high-dimensional features of coronary DSA images. Then, the high-dimensional feature differences of different frames in the coronary DSA image sequence are calculated, and non extremum suppression methods are introduced to obtain the heartbeat keyframes of the coronary DSA image sequence. We conducted experiments on a self-developed coronary DSA heartbeat keyframe recognition dataset and demonstrated the effectiveness and accuracy of the CDKD-w+ method. The contributions of this article are as follows:

(1) We propose a CDKD-w+ method based on deep learning that can accurately locate heartbeat keyframes in coronary DSA image sequences.
(2) We have demonstrated that pSp encoders encoded in the w+ space are suitable for tasks related to images captured by X-ray sensors, and demonstrated the potential of pSp encoders in locating the coronary artery itself rather than the background.
(3) We have demonstrated the effectiveness and accuracy of our proposed method through experiments.

## 2. Related Work

### 2.1. Coronary DSA Related Work

At present, research on auxiliary coronary DSA mainly includes various types of studies such as 3D modeling, calculation of quantitative blood flow fraction, detection of coronary stenosis, matching of DSA and CT, coronary segmentation, and localization of key frames in angiography. In 2009, Yang et al. proposed a method for 3D modeling of coronary arteries based on DSA imaging principles [21]. This method only requires two DSA images from different camera positions to complete the 3D modeling of coronary arteries, but the precision of the 3D model is limited and cannot reach the level of a camera. In 2021, Xu, Tu, and others demonstrated that their proposed quantitative blood flow fraction can accurately estimate blood flow reserve fraction and improve clinical outcomes [22]. QFR involves three-dimensional modeling of blood vessels, but requires manual annotation of key points for correction. In 2020, Zhang et al. proposed a stenosis detection method based on a multi view learning model [23], which can directly quantify the degree of coronary artery stenosis in intraoperative DSA images. In 2022, Wu et al. proposed a method of matching CT with DSA [24], which compensates for the shortcomings of DSA with the three-dimensional effect of CT. However, due to the static three-dimensional model of CT, this method has certain errors. In 2023, Zhang et al. proposed an image segmentation method that can segment coronary arteries in DSA images [25], thereby assisting doctors in determining coronary artery stenosis. In 2024, Zhang et al. proposed a convolutional long short-term memory network to achieve keyframe localization of coronary DSA image sequences [26]. This method defines frames filled with contrast as keyframes and focuses on determining the start and end frames of contrast filling.

The above methods assist the application of DSA in various ways, but do not consider the impact of heartbeat on DSA images. This article fills this gap and achieves the localization of heartbeat keyframes, providing an important tool for coronary 3D reconstruction.

### 2.2. Related Work on Three-Dimensional Modeling Based on Deep Learning

At present, deep learning-based 3D modeling has achieved camera level accuracy, and only requires a small number of perspective images to complete modeling work, with broad application prospects. In 2020, Mildenhall et al. proposed the NeRF method [19], which introduces the concept of neural radiation field and enables neural networks to perform voxel rendering based on a small number of perspectives of images, thereby achieving high-precision 3D modeling. In 2021, Gu et al. improved the NeRF method and proposed the StyleNeRF method [27], which combines the NeRF method with the StyleGAN method [28] to achieve the generation of 3D models by integrating NeRF’s 3D modeling ability and StyleGAN’s image generation ability. In 2022, Chan et al. proposed the EG3D method [29], which is based on StyleGAN2 [30]. They proposed a three plane reshape method that restructures the feature maps generated by the StyleGAN2 generator into three-dimensional features, and then models the three plane features using a neural renderer. In 2023, Bhattarai et al. proposed trilanet [31], which adds a correction module to EG3D. After EG3D training is completed, the difference between the original image and the generated image is calculated again, and the difference image is input into a three-plane codec to obtain an offset correction feature map. Combined with the EG3D feature map, a more accurate generated image can be obtained. In 2024, Khatib et al. proposed TriNeRFLet [32], which improves the three planes of EG3D by enabling the network to learn wavelet representations of feature planes rather than the feature planes themselves, thereby achieving higher modeling accuracy.

The core concept of the above method is to find a feature vector suitable for the current image in high-dimensional space, in order to achieve 3D modeling. However, this method has encountered difficulties in generalizing to test set samples. In response, the academic community has proposed GAN inversion networks to encode new samples, convert them to high-dimensional spaces, and then use the above modeling methods to generate 3D renderings. In 2021, Richardson et al. proposed the pSp method [20], which utilizes a feature pyramid to extract feature maps from images and encodes the feature maps in w+ space using the map2style module. In 2021, Tov et al. proposed the e4e encoder [33]. The logic of the e4e encoder is to simultaneously predict the w-space encoding of the image and the offset from w-space to w+ space through the encoder, thereby obtaining the latent code in w+ space. In 2024, Li et al. proposed an encoding method suitable for multi-objective scenes [34], which combines image segmentation with GAN inversion network to achieve synchronous encoding of target instances and backgrounds.

The above method is mainly used in the field of facial 3D modeling and has not been used in coronary artery DSA images. This paper introduces it for the first time in coronary artery DSA image 3D modeling, and uses pSp encoder to complete high-dimensional spatial encoding of the image, thereby assisting in the localization of heartbeat keyframes.

## 3. Method

**Figure 3:**
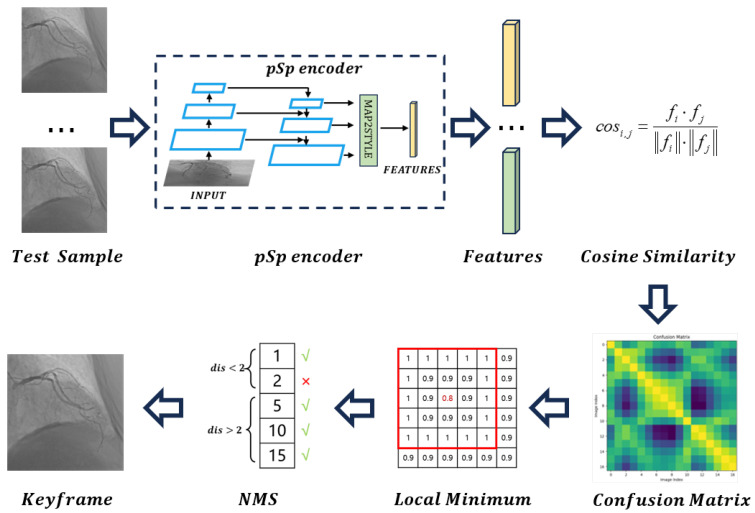
Overall framework of CDKD-w+. Different images are extracted features by the pSp encoder, then the cosine similarity between feature vectors is calculated, forming a confusion matrix based on cosine similarity. Keyframes are located by searching for local minimum in the confusion matrix.

In this section, we provide the implementation details of the CDKD-w+ method, which mainly includes two parts: pSp module pre training and heartbeat keyframe recognition. The operation mechanism of the CDKD-w+ method is shown in Figure 3.

### 3.1. Pre-Training Process of pSp Encoder

The w+ space is a high-dimensional space of 18 × 512 dimensions. Based on the StyleGAN2 image generation network, it can find an encoding in the w+ space and convert it into an image that is similar to the original image, thereby achieving image generation. The pSp encoder can transform new images that were not involved in training into an encoding in the w+ space. The generated code represents the high-dimensional features of the input image. The image generation network based on StyleGAN2 can reconstruct the test set samples based on the encoding in the w+ space.

In order to perform w+ space encoding on coronary DSA images, we simultaneously introduce adversarial generative networks and GAN inversion networks for 3D scenes. The purpose of 3D GAN is to find a w+ space latent code and output a 3D perception image with 3D effects. This method can generate 3D perception images, but when encountering new samples, the 3D GAN needs to be retrained. The function of GAN inversion network is to encode an image and convert it into a high-dimensional vector in w+ space. Combining GAN inversion network with 3D GAN can achieve end-to-end 3D perceptual image generation and obtain the 3D model of the input image. In the experiment, we chose a pre-trained EG3D network as the adversarial generation network for 3D scenes and pSp method as the GAN inversion network.

**Figure 4:**
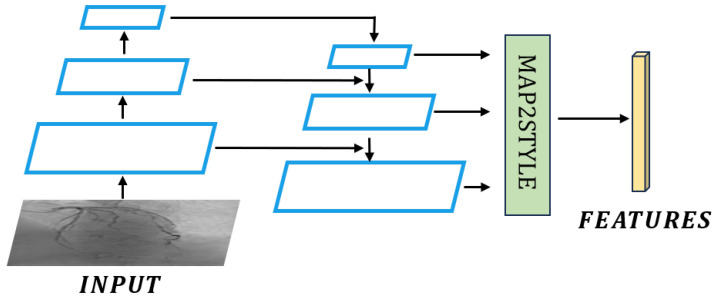
pSp schematic diagram.

The pSp encoder mainly consists of two parts: the feature pyramid module and the map2style module. When performing image inversion, the coronary artery DSA images from the test set are input into the feature pyramid module to obtain feature maps, which are then input into 18 map2style modules to obtain 18 dimensional features in the w+ space. The w+ space features can be input into EG3D to obtain a three-dimensional rendering of the test sample. The usage of pSp is shown in Formula (1), where pSp represents the pSp encoder, *f* represents the feature vector in w+ space, and *x* represents the image. The pSp schematic diagram is shown in Figure 4.

(1) $$f=pSp\left(x\right)$$

In the field of face generation, the loss function of the pSp encoder mainly consists of three parts: L2 loss, LPIPS loss, and ID loss. In Dynamic Style-based Augmentation (DSA), we have removed the ID loss, which is used to determine whether the image is a face. The loss function now comprises L2 loss and LPIPS loss, and the formula is as follows:

(2) $$L\left(x\right)=\alpha L_{2}\left(x\right)+\beta L_{LPIPS}\left(x\right)$$

(3) $$L_{2}\left(x\right)={∥x-pSp(x)∥}_{2}$$

(4) $$L\left(x\right)={∥F(x)-F(pSp(x))∥}_{2}$$

where *x* represents an image, psp(x) represents the generated image obtained by encoding the input image with pSp and decoding it with StyleGAN2, and *F* represents the pre-trained feature extractor used in the LPIPS calculation process.

We use a pre-trained EG3D model to obtain the labels required for training the pSp encoder. EG3D can convert encodings in the w+ space into generated images. During the training process of pSp, we use real images as inputs to the pSp encoder. The input of EG3D, the w+ space encoding, is used as the label for the pSp encoder, thus enabling the training of the pSp encoder. The trained pSp module can encode coronary artery DSA images into the w+ space, and the resulting latent code contains high-dimensional features, including the three-dimensional information of coronary artery DSA images.

### 3.2. Heartbeat Keyframe Localization Process

We use the pre-trained pSp module as a feature extractor to extract high-dimensional features of all samples in the w+ space of the coronary DSA image sequence to be tested, and calculate the cosine similarity between each image, as shown in the Formula (5).

(5) $$\cos_{i,j}=\frac{f_{i}\cdot f_{j}}{∥f_{i}∥\cdot ∥f_{j}∥}$$

where *x* represents the coronary DSA image, *f* represents the feature vector of the w+ space of the image, and $\cos_{i,j}$ represent the cosine similarity between the feature vector of the *i*-th image and the feature vector of the *j*-th image in the sequence. Cosine similarity can measure the degree of similarity between two vectors in direction. The closer it is to 1, the more similar the feature vectors are, which means that the two frames of the coronary DSA image sequence are more similar. In theory, as the heart beats, the coronary arteries undergo periodic vasodilation and contraction, with the greatest difference between the image at the most vasodilation moment and the image at the most vasoconstriction moment, resulting in the smallest cosine similarity.

**Figure 5:**
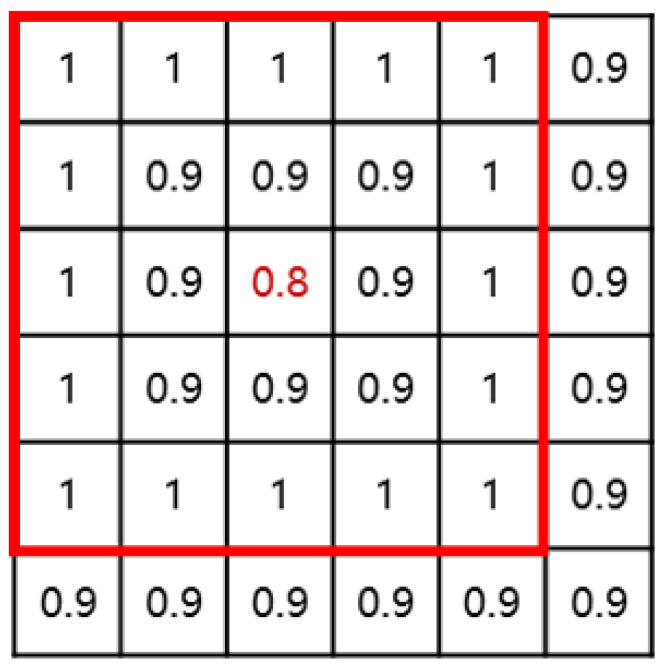
Illustration of obtaining a local minimum value.

We form a two-dimensional matrix based on the cosine similarity between each image, traverse the points in the matrix, compare the values of each point with those of adjacent points within a distance of 2, and select the point with the minimum value. The two images corresponding to this point are the two images with a large difference in cosine similarity, which most likely represent the most relaxed and contracted moments. Therefore, we will add these two images to the candidate sequence of heartbeat keyframes at the same time. The schematic diagram of this process is shown in Figure 5.

(6) $$Key\;f\;rame\;=\;min_{2*2}\left(Matrix\right)$$

**Figure 6:**
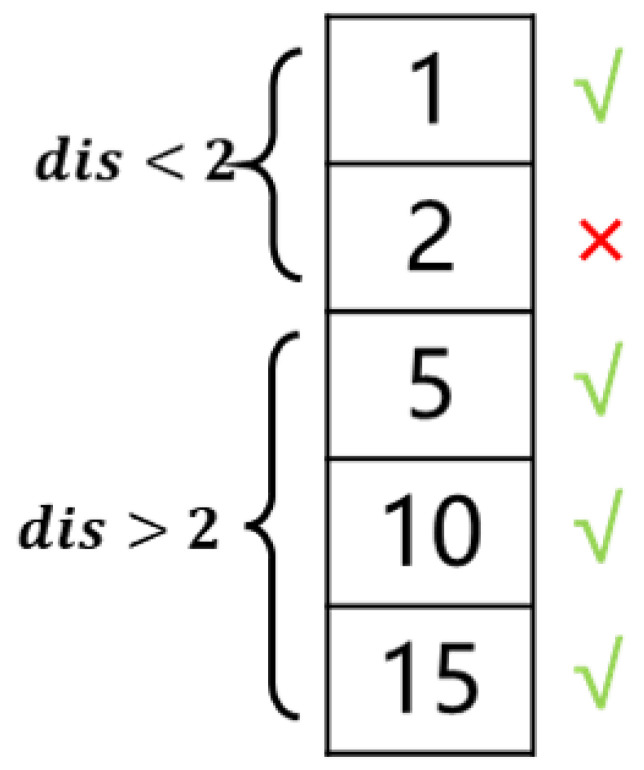
Illustration of non-minimum suppression. Assuming the 0th frame is a keyframe, the 1st frame within a distance of 2 frames will be suppressed, while keyframes with a distance greater than 2 frames will be retained.

To avoid the simultaneous occurrence of minimum values in adjacent frames, we introduce non-minimum suppression methods. Firstly, sort the values in the candidate sequence of heartbeat keyframes in ascending order, and then add the two frames corresponding to the minimum values to the final sequence of heartbeat keyframes. If the interval between the newly input frame and a frame in the sequence is less than 2, skip that frame. After traversal, all heartbeat keyframes in this sequence can be obtained. The schematic diagram of this process is shown in Figure 6.

## 4. Experiment and Analysis

### 4.1. Experimental Equipment

The experiment was conducted on a server equipped with an Intel Core i7 12700F CPU and two GeForce RTX 3080 Ti graphics cards, each with 12GB of memory. The server runs Ubuntu 20.04.4 with CUDA 12.2, Python 3.9, and PyTorch 2.1.2.

### 4.2. Dataset

Since there is currently no coronary DSA dataset serving the heartbeat keyframe recognition task, we collected some coronary DSA image sequences and annotated them to form the dataset. The coronary DSA sequences in our dataset are from a publicly available dataset collected by Danilov et al. [35], which is a coronary DSA stenosis detection dataset containing coronary DSA sequences from 100 patients. The clinical and demographic data of the study population are shown in Table 1.

**Figure 7:**
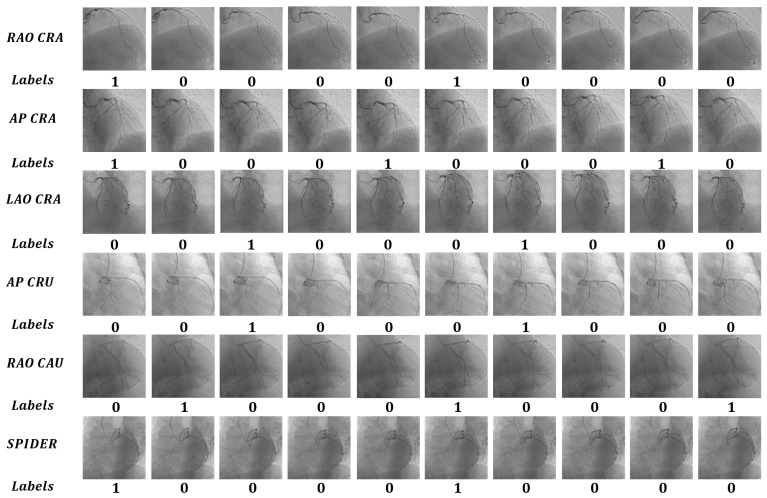
Dataset diagram. The dataset consists of two parts: images and labels. The images include sequences from multiple positions such as RAO CRA, AP CRA, etc. Each sequence has labels categorized as 1 or 0. In this context, 1 indicates that a particular image is a keyframe related to the heartbeat of that sequence, while 0 indicates it is not.

The dataset contains 50 sets of coronary DSA image sequences, each containing at least one complete heartbeat cycle. The dataset contains a total of 959 coronary DSA images. The dataset includes coronary DSA angiography image sequences from multiple machine positions, including right shoulder position (RAO CRA), head position (AP CRA), left shoulder position (LAO CRA), foot position (AP CRU), liver position (RAO CAU), spider position (Spider), etc., covering the main machine positions of coronary DSA angiography; the specific quantities are shown in Table 2. The dataset images are grayscale images, and we uniformly set the image size of each sequence to 512 × 512. We annotated each image in the dataset, setting the label of the image when the heartbeat contracted or relaxed to the pole to 1, representing it as a heartbeat keyframe, while setting the label of the remaining images to 0, representing non-heartbeat keyframes. The dataset sequence and labels are shown in Figure 7. In the establishment of the dataset, we followed a methodology involving annotation by two individuals and verification by one individual. In cases of disagreement, three individuals voted on the contentious item to determine the final annotation for that data.

### 4.3. Comparative Methods

To demonstrate the effectiveness of our method, we compared it with three image difference evaluation methods, namely L1 loss, PSNR, and SSIM, to demonstrate the effectiveness and necessity of our heartbeat keyframe recognition method. We use L1 loss, PSNR, and SSIM methods to calculate the differences between each image in the coronary DSA image sequence. In theory, the difference between the most relaxed and contracted frames of the coronary artery is the largest, so the two frames with the largest difference can be selected as heartbeat keyframes. In terms of implementation details, similar to our proposed heartbeat keyframe recognition method, the local extremum in the inter frame difference matrix is first selected. The L1 loss method selects the maximum value, while the PSNR and SSIM methods select the minimum value. Then, the calculated frames are subjected to non extremum suppression with an interval of 2 frames to determine the heartbeat keyframe.

#### 4.3.1. L1 LOSS

L1 loss is a pixel level image difference assessment method that calculates the L1 loss by summing the absolute differences of each pixel in two images. The L1 loss formula is as follows:

(7) $$L1=\sum_{p\in P}\left|x\left(p\right)-y\left(p\right)\right|$$

where *p* represents each pixel in the image, and *x* and *y* represent two images in the same sequence, respectively.

#### 4.3.2. PSNR

Peak Signal-to-Noise Ratio (PSNR) is an indicator used to quantify image quality, the higher the PSNR value, the smaller the difference between the two images. The PSNR formula is as follows:

(8) $$MSE=\frac{1}{mn}\sum_{i=1}^{m}{\sum_{j=1}^{n}\left[I\left(i,j\right)-K\left(i,j\right)\right]}^{2}$$

(9) $$PSNR=10\cdot \log_{10}\left(\frac{MAX^{2}}{MSE}\right)$$

where MSE is calculated from the difference between two images, where *m* and *n* represent the number of rows and columns of the images, and *I* and *K* represent the two images. In this context, *i* and *j* denote the coordinates of pixels in the images, and $MAX^{2}$ represents the square of the maximum possible pixel value in the images.

#### 4.3.3. SSIM

The Structure Similarity Index Measure (SSIM) is an image difference evaluation method that comprehensively considers the differences in pixel values and structural information between images. It takes into account the brightness, contrast, and structure of the images. The smaller the SSIM value, the greater the difference between the two images. The SSIM formula is as follows:

(10) $$SSIM\left(x,y\right)=\frac{\left(2\mu_{x}\mu_{y}+C_{1}\right)\left(2\sigma_{xy}+C_{2}\right)}{\left({\mu_{x}}^{2}+{\mu_{y}}^{2}+C_{1}\right)\left({\sigma_{x}}^{2}+{\sigma_{y}}^{2}+C_{2}\right)}$$

where $\mu_{x}$, $\mu_{y}$ are the mean values of images *x* and *y*, respectively. $\sigma_{x}$, $\sigma_{y}$ are the variance of images *x* and *y*. $\sigma_{xy}$ is the covariance. $C_{1}$ and $C_{2}$ are two constants used to avoid division errors.

#### 4.3.4. ResNet

We conducted a comparative experiment using the ResNet18 network as a feature extractor. We built an image classification network with a ResNet18 backbone to distinguish between the left and right coronary arteries. The model achieved an accuracy of 99.6% in classifying the left and right coronary arteries. We used the trained ResNet18 to extract image features, replacing the feature vectors extracted by pSp. Subsequently, we calculated the cosine similarity between the feature vectors of different images extracted by ResNet18 to maintain consistency with the following methods.

### 4.4. Experimental Results and Discussions

We measure the differences between each heartbeat keyframe recognition method and the real situation of the dataset through three indicators: accuracy, precision, and recall. The results are shown in the Table 3.

From the experimental results, it can be seen that our method can accurately identify heartbeat keyframes, while L1 loss, PSNR, and SSIM perform poorly in the task of identifying heartbeat keyframes.

**Figure 8:**
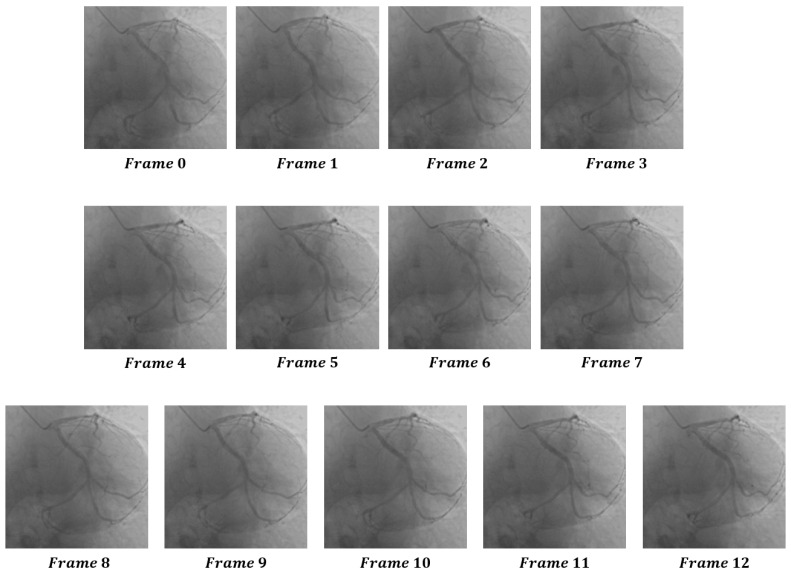
A complete DSA sequence designed to visually demonstrate the actual performance of different methods for recognizing heartbeat keyframes within a unified sequence.

**Figure 9:**
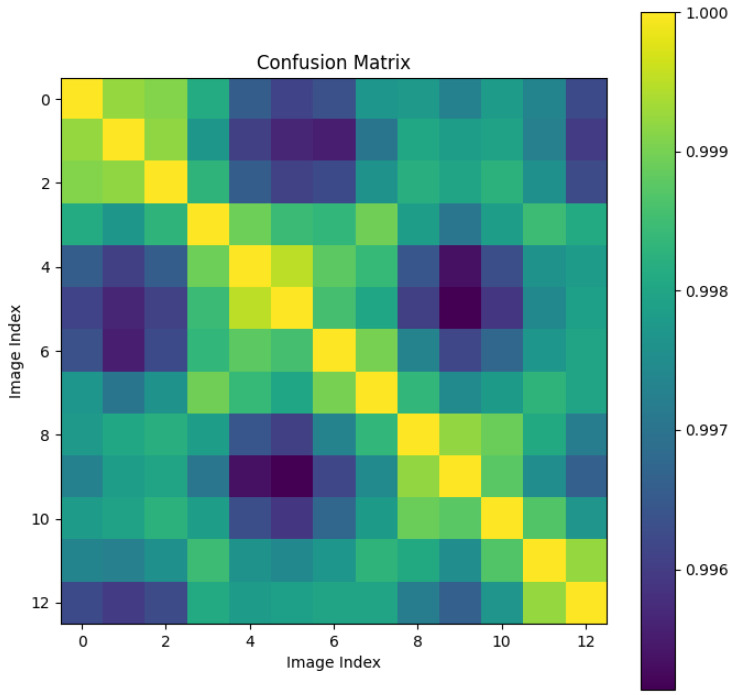
CDKD-w+ confusion matrix.

**Figure 10:**
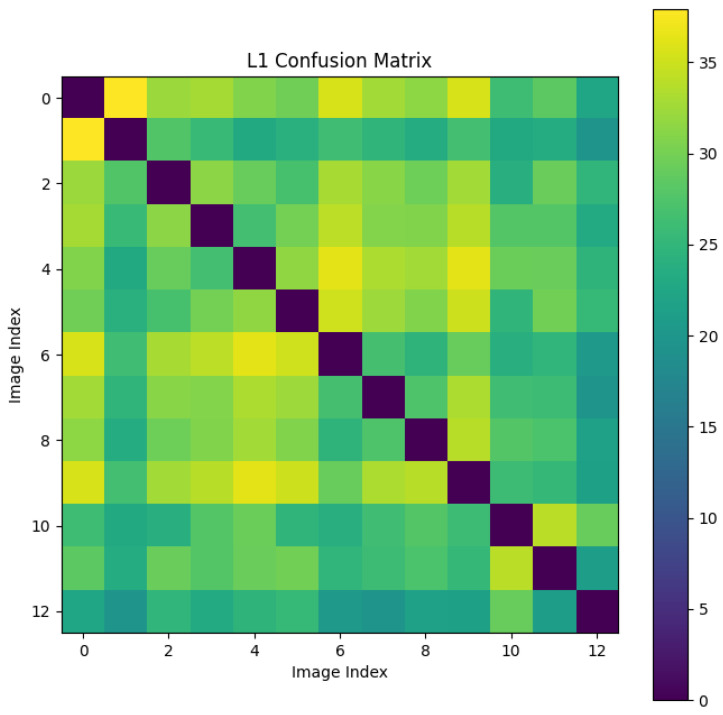
L1 LOSS confusion matrix.

**Figure 11:**
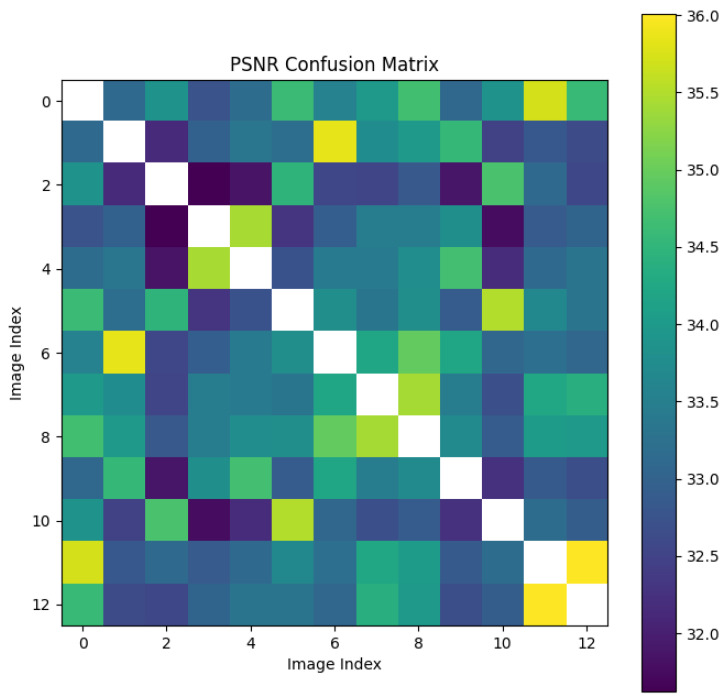
PSNR confusion matrix.

**Figure 12:**
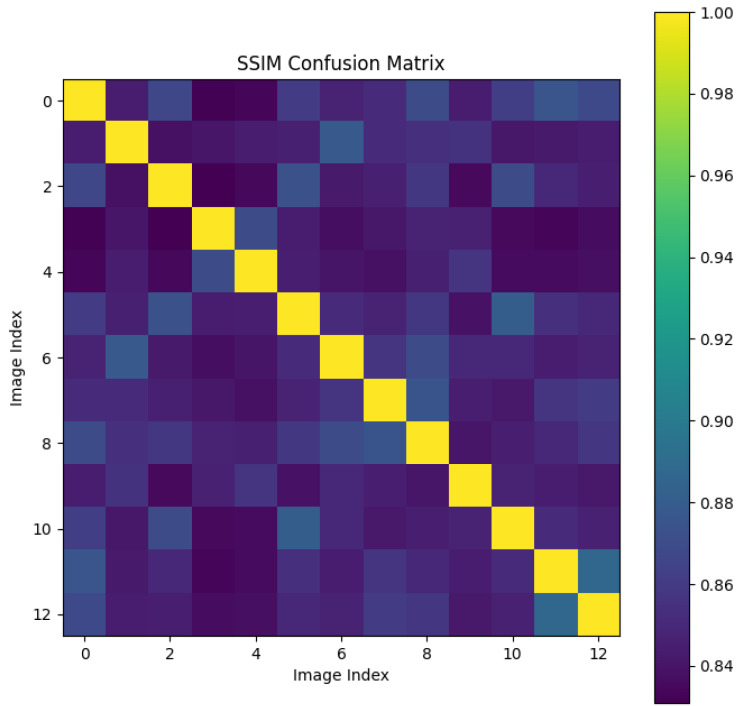
SSIM confusion matrix.

**Figure 13:**
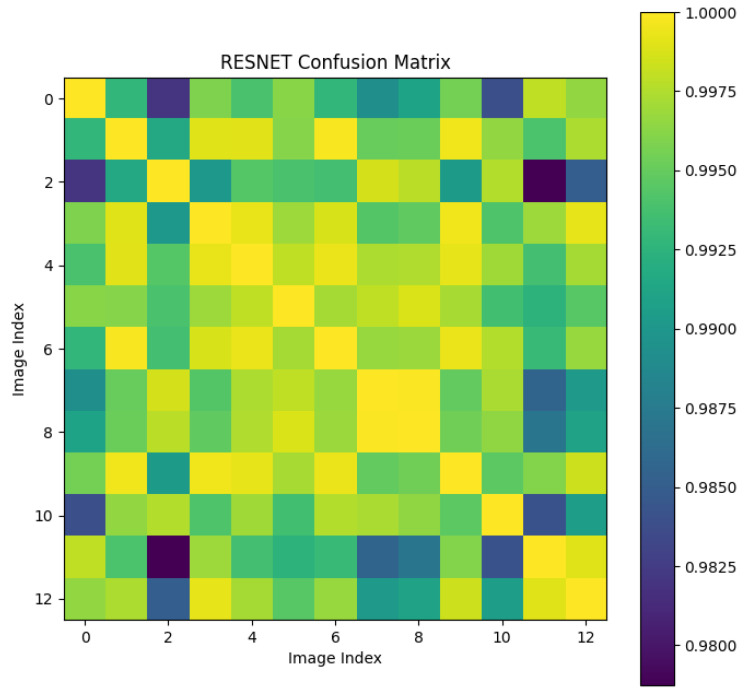
ResNet confusion matrix.

We randomly selected a coronary DSA image sequence from the dataset, as shown in Figure 8. We used our method, L1 loss, PSNR, and SSIM methods to calculate the inter frame differences, and plotted the difference values between each frame as a confusion matrix, as shown in Figure 9, Figure 10, Figure 11, Figure 12 and Figure 13. From the figure, it can be seen that our method can locate the heartbeat keyframe and reflect the heartbeat cycle, while the comparative method cannot accurately locate the heartbeat keyframe.

**Figure 14:**
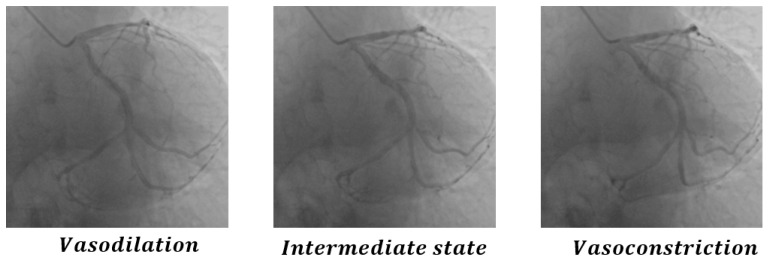
Illustration of vasodilation frame, intermediate frame and vasoconstriction frame.

We select the coronary vasodilation frame, intermediate frame, and vasoconstriction frame from the sequence in the figure, and calculate the difference between the figures, as shown in Figure 14, the specific values are shown in Table 4. Theoretically, if all methods can sense coronary artery differences, the difference between the vasoconstriction frame and the vasodilation frame is the largest; the values of CDKD, PSNR, SSIM, and ResNet are the smallest, and L1 LOSS is the largest. As can be seen from Figure 7, there are differences in coronary arteries between frames, which can be easily perceived by human eyes. However, L1 loss, PSNR, SSIM, and ResNet fail to pay attention to the coronary artery itself, resulting in poor positioning effect of heartbeat key frames, while our method focuses on the coronary artery, thus achieving better results and accurately positioning heartbeat key frames.

The experimental results show that the key point of heartbeat key frame judgment is to make the method pay attention to the coronary artery itself rather than the background, and the high-dimensional features in w+ space extracted by pSp method as a feature extractor effectively retain the information of coronary artery and suppress the background interference information to a certain extent, so as to realize accurate heartbeat key frame recognition.

## 5. Conclusions and Discussion

We provide a heartbeat key frame identification method for 3D modeling of coronary DSA image sequence obtained by X-ray sensor, which can accurately locate the diastolic and systolic frames and reduce the influence of heartbeat on the accuracy of 3D modeling of coronary DSA. In addition, we have proved the superiority of using a GAN inversion network such as PSP encoder as encoder to encode images, and demonstrated the potential of encoding in w+ space corresponding to styleGAN2 through experiments. In the future, we can use this feature to encode the sensor image in high-dimensional space by using a three-dimensional encoder, so as to realize image difference detection without special labeling. The focus of the method used in this study is to locate keyframes of heartbeats. However, the method in this study is only suitable for calculating the size of differences between paired samples, making it difficult to directly locate vasoconstriction and vasodilation frames. In the future, methods like image segmentation can be introduced to independently locate vasoconstriction and vasodilation frames.

## Figures and Tables

**Table 1:** Clinical and demographic data of the study population [35].

Parameter	Value
Total number of patients	100
Mean age ± SD, years	60.3 ± 13.8
Men, *n* (%)	68 (68%)
Women, *n* (%)	32 (32%)
Body mass index (kg/ $m^{2}$)	21.6 ± 5.1
Diagnosis	CAD
Class I NYHA	5 (5%)
Class II NYHA	84 (84%)
Class III NYHA	11 (11%)
Arterial hypertension	53 (53%)
Diabetes mellitus	14 (14%)
Chronic heart failure, classes 1–2	36 (36%)
Coronary artery stenosis > 70% (*n*, %)	100 (100%)

**Table 2:** Dataset position distribution.

Position	Number
RAO CRA	16
AP CRA	5
LAO CRA	4
AP CRU	15
RAO CAU	5
SPIDER	5

**Table 3:** Comparative experimental results.

Metric	Ours	L1	SSIM	PSNR	ResNet
Accuracy	0.97	0.63	0.64	0.65	0.67
Precision	0.93	0.20	0.23	0.23	0.30
Recall	0.95	0.23	0.26	0.26	0.37

**Table 4:** Comparative experimental results in vasodilation frame, intermediate frame, and vasoconstriction frame in Figure 14.

Metric	Vasodilation & Intermediate	Vasodilation & Vasoconstriction	Intermediate & Vasoconstriction
CDKD-w+	0.998	0.996	0.998
L1 LOSS	26.11	22.39	29.10
PSNR	33.86	34.58	32.92
SSIM	0.862	0.878	0.846
ResNet	0.984	0.997	0.991

## Data Availability

Data can be obtained from the public dataset in [35].
